# Nadofaragene firadenovec: a breakthrough in the field of bladder oncology

**DOI:** 10.3389/fruro.2023.1206398

**Published:** 2023-09-15

**Authors:** Abdullah Nadeem, Khulud Qamar, Wajeeha Bilal, Laiba Imran Vohra, Areeba Ahsan, Rabeea Tariq

**Affiliations:** ^1^ Department of Medicine, Dow University of Health Sciences, Karachi, Pakistan; ^2^ Department of Medicine, Ziauddin University, Karachi, Pakistan

**Keywords:** Nadofaragene firadenovec, BCG-unresponsive non-muscle invasive bladder cancer, gene therapy, interferon-2b, carcinoma in situ, immunostimulatory, antiangiogenic

## Abstract

Muscle-invasive bladder tumors pose a grave mortality risk due to their propensity for distant metastases. The therapeutic spectrum for such tumors encompasses surgery, chemotherapy, and radiation, tailored to the cancer’s severity. In the context of high-risk Bacillus Calmette-Guérin (BCG)-unresponsive non-muscle invasive bladder cancer (NMIBC), a novel treatment has emerged as a beacon of hope. Nadofaragene firadenovec, a pioneering gene therapy, has gained worldwide approval for combating this condition, marking a watershed moment in bladder cancer therapy. Nadofaragene firadenovec is ingeniously designed to address high-risk BCG-unresponsive NMIBC, particularly carcinoma *in situ* (CIS) with or without papillary tumors, in adult patients. Rooted in a vector DNA, this therapy encodes interferon (IFN)-2b, which imparts urothelial cells with the ability to generate IFN-2b. The resulting cascade of events triggers a multifaceted assault on cancer, characterized by its immunostimulatory, antiangiogenic, and apoptotic effects. The therapeutic efficacy of nadofaragene firadenovec rests on its capacity to exploit the transformed urothelial cells to deliver these targeted anticancer activities. The evolutionary trajectory of nadofaragene firadenovec culminated in its monumental approval in December 2022 by the United States, signifying a pivotal juncture in the field. Notably, a segment of patients, approximately 30%, prove refractory to BCG treatment. For these individuals, alternative therapeutic avenues are imperative. Presently, the landscape for patients with non-muscle invasive bladder cancer lacks a definitive, enduring solution. Against this backdrop, the introduction of nadofaragene firadenovec heralds a momentous stride toward the global availability of an authorized therapeutic intervention.

## Background

Muscle-invasive bladder tumors carry a high risk of mortality due to distant metastases (1). Treatment can range from surgery, and chemotherapy to radiation therapy, depending on the severity of the cancer. In some cases, a combination of treatments may be recommended to most effectively treat cancer. With an estimated 1.6 million people affected worldwide in 2018, bladder cancer was a major global health concern. This figure included 549,000 new cases and 200,000 deaths. Rates of bladder cancer were highest in Southern and Western Europe at 15 cases per 100,000 people, followed by North America at 15, 13, and 12 cases per 100,000 people. Northern Africa and Western Asia experienced the highest rates of bladder cancer deaths, followed by Southern Europe (2). About 90% of bladder cancer diagnoses occur in people aged 55 and over, and the disease is four times more common in males than females (3). Approximately 550,000 new cases of bladder cancer are diagnosed each year, making it one of the world’s top ten most common cancer types (4).

About 20-40% of bladder cancer cases are non-muscle invasive bladder cancer (1) in which the malignant cells typically form flat, papillary lesions on the bladder wall. These papillary tumors can grow in size or number and have the potential to spread to other organs in the body which can lead to invasive bladder cancer, a more serious form of the disease. The most common treatment for NMIBC is Bacillus Calmette-Guérin (BCG) immunotherapy, which can provide long-term remission in up to 70% of patients. However, a subset of patients does not respond to BCG treatment, considering it to be more aggressive and difficult to treat than other types of bladder cancer. While the cause of BCG-unresponsive NMIBC is not known, it is thought to be due to a combination of genetic and environmental factors.

The immune system recognizes and eliminates foreign invaders and cancer cells. In NMIBC, the tumor cells can evade the immune system, allowing them to grow and proliferate. BCG immunotherapy is used to stimulate the immune system to recognize and destroy bladder cancer cells. The BCG vaccine is administered directly into the bladder, where it triggers an inflammatory response that stimulates the immune system to attack tumor cells. This therapy is effective in up to 70% of NMIBC cases. However, the remaining 30% of patients are unresponsive to BCG treatment. These patients may require alternative treatments, such as chemotherapy and radiation therapy, to manage their disease.

## Pathophysiology of BCG-unresponsive NMIBC

In cases with BCG unresponsive non-muscle invasive bladder cancer (NMIBC), the Bacillus Calmette-Guérin (BCG) treatment is unable to successfully treat the malignancy. The features of the tumor, such as its bigger size, higher grade, multifocality, and presence of *in situ* carcinoma, frequently have a role in its resistance to BCG therapy. The immune system’s reaction, which includes TLRs and CD4+ and CD8+ T cells, is also important in the ineffectiveness of BCG. Immune tolerance strategies can prevent immune recognition and destruction, such as by expressing immune checkpoint molecules like PD-L1. BCG resistance can also result from genetic alterations, such as those in the mitogen-activated protein kinase (MAPK) pathway. BCG response may also be impacted by the tumor microenvironment, which is made up of several cell types, extracellular elements, and signaling chemicals. In this microenvironment, regulatory T cells and myeloid-derived suppressor cells provide an immunosuppressive environment that impairs the immune response and promotes tumor progression (5). BCG resistance is also influenced by host-related variables such as age, general health state, and immunological capability. Patients who have immune systems that are impaired by underlying illnesses or immunosuppressive drugs are more likely to show decreased response to BCG treatment. Treatment results may also be affected by variations in the BCG strain utilized or the delivery method (6). To improve treatment outcomes for patients who do not respond to standard BCG therapy, new therapeutic approaches, such as immune checkpoint inhibitors, targeted therapies, or combinations, must take into account the complex pathophysiological mechanisms underlying BCG unresponsive NMIBC.

## Current treatment options

**Figure 1:**
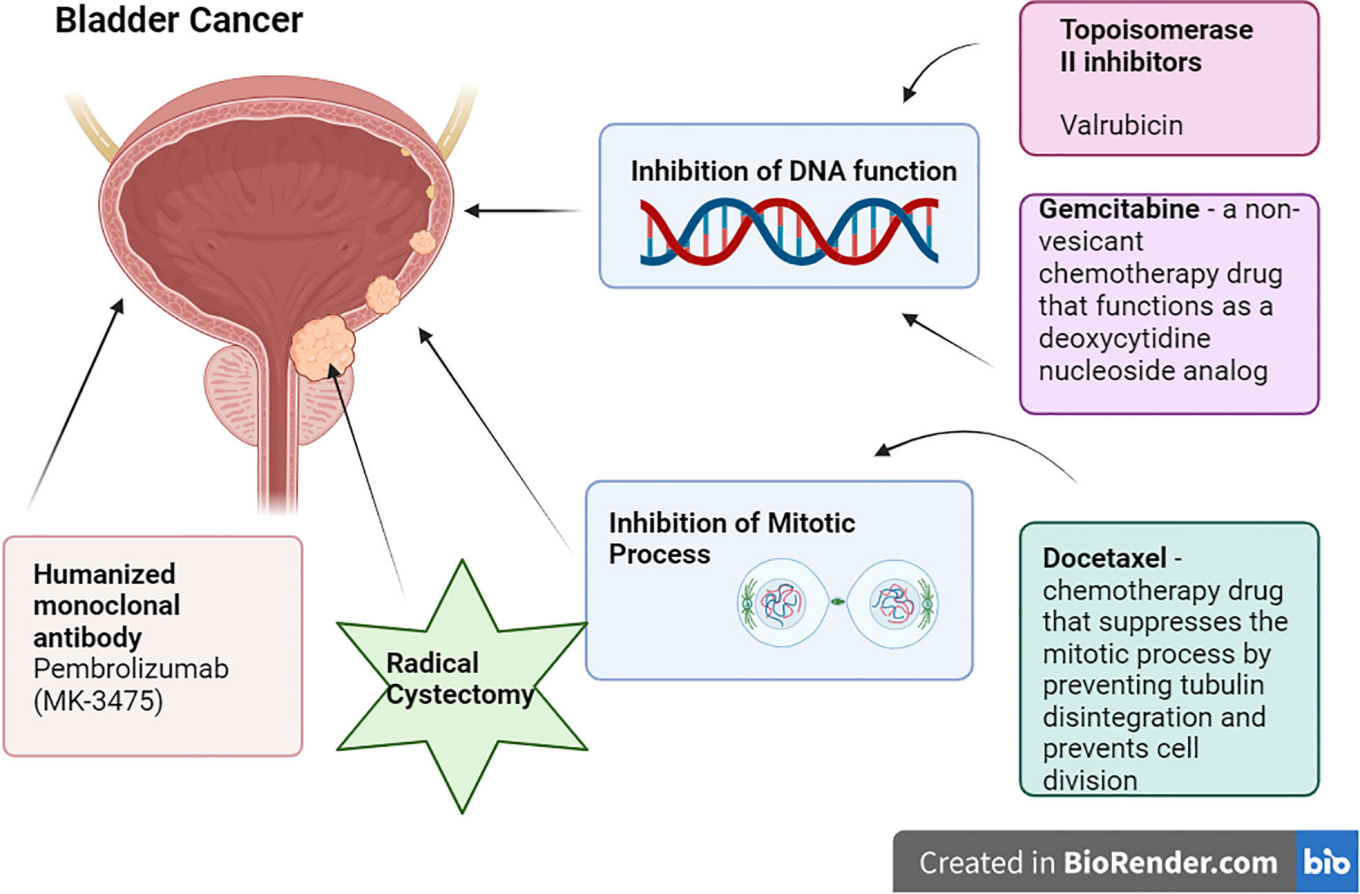
Current Treatment Options for NMIBC (created from Biorender.com).

1. Radical Cystectomy: For patients with high-risk, BCG-unresponsive, and for aggressive or muscle-invasive bladder cancer, radical cystectomy is the gold standard therapy, providing efficient care and long-term disease management (7). It lessens the chance of development and recurrence by removing the malignant bladder and surrounding tissues. The operation also aids in determining the stage, relieving symptoms, and removing lymph nodes, preventing cancer from spreading to other body areas. The bladder and lymph nodes must be removed in order to make treatment choices after the operation (8). The major operation known as a radical cystectomy entails medical risks, a loss of bladder function, emotional effects, and a protracted recovery period. It may result in urine leakage, urethral narrowing, urinary tract infections, and problems with urinary diversion. To adjust to the changed urinary system, patients may need to change their regular habits, hydration consumption, and food. Urinary leaks, strictures, urinary tract infections, and problems with urine diversion are possible postoperative consequences. In order to adjust to the changed urinary system, lifestyle adjustments could also be required, which would make some activities more difficult. Due to changes in anatomy and body image, radical cystectomies can influence sexual performance. For males, the removal of the bladder may necessitate the removal of the prostate gland, which might have an impact on erectile function. Long-term observation is necessary for further complications and recurrence (9) (**Figure 1**).

2. Valrubicin: Valrubicin (second-generation anthracycline) is a cell cytostatic medication that produces its antitumorigenic action by contacting the bladder wall and then being absorbed by cancer cells, where it exerts its cytostatic effect, reducing the growth of tumoral cells (10). According to the FDA, patients for whom urgent cystectomy would be associated with unacceptable morbidity or death are expressly mentioned as candidates. The acceptance of valrubicin in modern treatment has been very slow due to its relatively restricted indication and subpar overall long-term effectiveness as salvage therapy (7).

3. Gemcitabine/Docetaxel: Gemcitabine is a non-vesicant chemotherapy drug that functions as a deoxycytidine nucleoside analog. It prevents the production of DNA, which causes cell death. Whereas Docetaxel is a chemotherapy drug that suppresses the mitotic process by preventing tubulin disintegration and prevents cell division (11). According to a 2019 retrospective research, Relapse Free Survival rates were 60% and 46% at 1 and 2 years, respectively, with 15.6% of patients advancing to Radical Cystectomy at a median duration of 11.3 months following induction (7).

4. Pembrolizumab: Pembrolizumab (MK-3475) is a humanized monoclonal antibody that binds to the PD-L1 receptor on immunological (T) cells, blocking them from engaging with their ligands (12). In a phase 2 trial, 18.4% of people had immune-related adverse events (AEs), and 1 treatment-related death from colitis following the drug (7) (**Table 1**).

**Table 1:** Comparative Overview of Treatment Modalities for Non-Muscle Invasive Bladder Cancer.

Treatment	Mechanism	Efficacy	Side Effects	Notes
**Radical Cystectomy**	Surgical removal of the bladder and surrounding tissues.	Efficient disease control, and long-term management.	High surgical risks, loss of bladder function, lifestyle adjustments.	The gold standard for high-risk cases. Requires careful patient selection.
**Valrubicin**	Second-generation anthracycline chemotherapy drug.	Limited overall long-term effectiveness, salvage therapy.	Cell cytostatic effect, bladder contact.	Indicated for cases where cystectomy risks are unacceptable.
**Gemcitabine/Docetaxel**	Chemotherapy drugs inhibiting DNA and cell division.	Relapse-free survival rates, induction treatment.	Cell death induction, reduced tumor growth.	Used as an alternative for selected patients, combined therapy.
**Pembrolizumab**	Humanized monoclonal antibody targeting PD-L1.	Immune response activation, durable response.	Immune-related adverse events.	Immunotherapy option for specific patients. Consider biomarker testing.
**Nadofaragene Firadenovec**	Gene therapy delivering IFN-2b.	Positive complete response rates, breakthrough therapy.	Bladder spasms, hematuria, urgency.	Emerging gene therapy with promising results. Intravesical administration.

## Nadofaragene as an adjunct therapy

There is an imperative need for innovative medicines in the treatment of NMIBC because of the high rates of progression and recurrence associated with existing therapy, and the significant side effects and morbidity associated with definitive therapies. Nadofaragene firadenovec (nadofaragene firadenovec-vncg; Adstiladrin^®^) is a Ferring Pharmaceuticals-developed non-replicating adenoviral vector-based gene therapy for the treatment of high-risk BCG-unresponsive NMIBC (13). The recombinant adenovirus Nadofaragene firadenovec delivers human interferon-cDNA to the bladder epithelium together with Syn3, a polyamide surfactant that speeds up viral transduction of the urothelium. Local interferon production causes tumor regression (7). In December 2022, nadofaragene firadenovec gained its first worldwide approval in the United States for the treatment of high-risk BCG-unresponsive NMIBC with carcinoma *in situ* (CIS) with or without papillary tumors in adults (13, 14). It is the first gene therapy to be approved for the treatment of bladder cancer (15). Individuals with immunocompromised and immunodeficient conditions should not administer nadofaragene firadenovec because it may spread disseminated adenovirus infection (16). Multiple trials were conducted which were efficacious, with a positive benefit-to-risk ratio. In a study conducted between 2016 and 2019, 25 (45%) of 55 patients with carcinoma *in situ* had their response sustained at 12 months. After the 103 patients who participated, 55 (53%) achieved a full response within 3 months of the first dosage, making it a breakthrough drug (17) (**Table 2**).

**Table 2:** Nadofaragene Firadenovec: Drug Information and Effects.

**Other Names**	Also referred to as Adstiladrin®, Instiladrin, nadofaragene firadenovec-vncg, rAd-IFNa/Syn3, SCH-721015, TR-002
**Drug Category**	Belongs to antineoplastic and IFNA2B gene therapy classes
**How It Works**	Operates through gene transfer, stimulating IFNA2B expression
**Administration**	Given intravesically (for bladder cancer) or intrapleurally (for mesotheliomas)
**Effect on Body**	Triggers interferon production post intravesical/intrapleural use; Stronger anti-vector immune responses tied to better outcomes in bladder cancer cases
**Absorption**	Across two trials, vector DNA was detected in blood (one patient) and urine (1/4 patients and 16/19 patients in separate trials)
**Common Side Effects**	Grade 1 or 2: Catheter-related discharge, fatigue, bladder spasms, urgent urination, chills, painful urination, fever Grade 3: Urgent urination, bladder spasms, fainting, high blood pressure, urinary incontinence

## Therapeutic trials

In a phase III clinical trial involving patients with BCG-unresponsive non-muscle invasive bladder cancer (NMIBC), characterized by an open-label, single-arm design and conducted across multiple centers, a remarkable complete response (CR) rate of 53.4% (95% CI 43.3%–63.3%) was achieved among a cohort of 103 patients with carcinoma *in situ* (CIS) [primary endpoint] (ClinicalTrials.gov identifier: NCT02773849) (17). Notably, this CR rate significantly surpassed the predetermined rate of 27% established for this specific cohort (p < 0.0001). The study enrolled 157 participants aged 18 years or older, possessing an Eastern Cooperative Oncology Group status of 2 or lower. These individuals received intravesical treatment with nadofaragene firadenovec at a concentration of 3 × 1011 viral particles/mL in 75 mL doses. Subsequent doses were administered at 3, 6, and 9 months for patients who exhibited an absence of high-grade recurrent disease. Exclusion criteria encompassed conditions such as upper urinary tract disease, urothelial carcinoma within the prostatic urethra, lymphovascular invasion, micropapillary bladder cancer, and hydronephrosis stemming from T1 disease.

In a phase II clinical trial carried out across multiple centers and employing an open-label design, patients diagnosed with BCG-refractory or relapsed non-muscle invasive bladder cancer (NMIBC) were investigated. The primary endpoint of interest pertained to the 12-month relapse-free survival rate in cases of high-grade disease. Among the study participants, a cohort of 21 individuals received a low dose (1 × 1011 viral particles/mL) of nadofaragene firadenovec, while another group of 19 patients received a higher dose (3 × 1011 viral particles/mL) of the same therapy [ClinicalTrials.gov identifier: NCT01687244] (18). Impressively, the observed 12-month relapse-free survival rate was 33.3% for those who received the low dose and 36.8% for those who received the high dose. In this trial, all patients were subjected to a single intravesical administration of nadofaragene firadenovec, infused in a 75 mL volume. Patients who remained free from recurrence of high-grade disease subsequently underwent additional treatment sessions with nadofaragene firadenovec at months 4, 7, and 10 (18).

In phase I studies, a phase Ib trial observed that 29% (2 out of 7) of patients with BCG-refractory NMIBC achieved a complete response (CR) when subjected to intravesical treatment involving nadofaragene firadenovec at a concentration of 3 × 1011 viral particles/mL in a 75 mL volume. The primary focus of this trial was to assess the transfection efficacy of a second dose (19). Another phase I trial explored intravesical treatment with nadofaragene firadenovec at concentrations ranging from 3 × 109 to 3 × 1011 viral particles/mL in a 75 mL volume. Within this trial, a complete response was attained in 41% (7 out of 17) of patients with BCG-refractory NMIBC. The primary objective of this study was centered around safety assessment. These findings highlight the potential efficacy of nadofaragene firadenovec as a therapeutic avenue for BCG-refractory NMIBC in phase I trials (20) (**Tables 3**, **4**).

**Table 3:** Clinical Trials Involving Nadofaragene Firadenovec for Various Indications.

Drug(s)	Indication	Phase	Status	Location	Identifier	Sponsor
Nadofaragene firadenovec	Bladder cancer	III	Active, not recruiting	USA	NCT02773849, rAd-IFN-CS003	Ferring Pharmaceuticals
Nadofaragene firadenovec	Bladder cancer	II	Completed	USA	NCT01687244, rAd-IFN-CS002	FKD Therapies Oy
Nadofaragene firadenovec	Bladder cancer	Ib	Completed	USA	NCT01162785	M.D. Anderson Cancer Center
Nadofaragene firadenovec	Bladder cancer	I	Completed	USA	NCT00536588	Merck Sharp & Dohme LLC
Nadofaragene firadenovec, celecoxib, gemcitabine	Mesothelioma	III	Active, not recruiting	Global	NCT03710876, EudraCT2017-003169-82, rAd-IFN-MM301	Trizell Ltd.
Nadofaragene firadenovec, celecoxib, chemotherapy	Mesothelioma	Ia	Completed	USA	NCT01119664	Abramson Cancer Center of the University of Pennsylvania
Nadofaragene firadenovec	Mesothelioma	I	Completed	USA	NCT01212367	Abramson Cancer Center of the University of Pennsylvania

**Table 4:** Results from Phase III Clinical trial.

Clinical Trial	Phase	Patient Population	Registry number	Outcome
Intravesical Nadofaragene Firadenovec gene therapy for BCG-unresponsive non-muscle-invasive bladder cancer: a single-arm, open-label, repeat-dose clinical trial (17).	III	157	NCT02773849	Complete response in 53.4% of patients with high-grade, BCG-unresponsive NMIBC for 3 months. Among these, 45.5% had a response maintained for 12 months.
MP16-01 Efficacy of Intravesical Nadofaragene Firadenovec for patients with carcinoma in situ (CIS), BCG-unresponsive non-muscle invasive bladder cancer (NMIBC): longer-term follow-up from the Phase III trial (14)	III	103	NCT02773849	24 months after the initial dose, 20/103 (19.4%) of the patients were still clear of HG recurrence. 20(36.4%) of the 55 CIS-Ta/T1 patients who received treatment and were able to obtain a CR did so at 24 months, and the average time of the HGRFS was 12.2 months. With a KM cystectomy-free survival of 64.6% (95% CI 54.1-73.3) and overall survival of 94.4% (95% CI 87.0-97.6) by 24 months, 33/103 (32%) of the patients had received cystectomies.
Antiadenovirus Antibodies Predict Response Durability to Nadofaragene Firadenovec Therapy in BCG-unresponsive Non–muscle-invasive Bladder Cancer: Secondary Analysis of a Phase 3 Clinical Trial (21).	III	91	NCT02773849	On assessing post-treatment serum antibodies, 42 pts (89%) were observed with peak post-treatment titers >800 compared to 26 (59%) of the nonresponses. Additionally, peak post-treatment titers >800 and peak antibody fold change >8 were present in 22 (47%) responders as opposed to eight (18%) nonresponses. Higher titers indicate a well-tolerance of the drug.

## Dosage, administration, and side effects

Nadofaragene (rAd-IFN/Syn3) is a gene therapy used for the treatment of bladder cancer. A single intravesical instillation of Nadofaragene, which contains x10 ^11^ viral particles, is administered every 3 months for a total of four doses (17). Nadofaragene is administered via intravesical instillation, which involves the injection of the therapy directly into the bladder. It acts on tumor cells and expresses INF alpha-2b intracellularly which further indirectly activates antiviral, antiproliferative, antitumor and immune-modulating effects (5). The most commonly reported side effects of Nadofaragene therapy include bladder spasms, hematuria, micturition urgency, fatigue, and urinary tract infection (17) (14). In the phase III clinical trial, the safety population included all treated patients in the CIS and the high-grade Ta or T1 illness groups. Sixty-six percent of patients had drug-related adverse events (AEs) of grade 1 or 2 whereas four percent of patients suffered grade 3 AEs. Grade 4 or 5 AEs were not documented. The most frequent grade 1 or 2 TRAEs (incidence 10%) were pyrexia (incidence 10%), weariness (incidence 20%), bladder spasm (incidence 15%), micturition urgency (incidence 14%), chills (incidence 12%), and discharge surrounding the catheter during instillation (incidence 25%). Urinary incontinence, bladder spasm, syncope, hypertension, and urgency to urinate (all two patients; incidence 1%), as well as syncope and syncope-like symptoms, were all grade 3 drug-related adverse events that were observed during the experiment (17). These side effects are generally mild to moderate in severity and resolve within a few days to weeks. In addition, Nadofaragene therapy may cause serious adverse events, including bladder contracture, urinary fistula, and bladder perforation, although these events are rare. Patients should be closely monitored for signs of adverse events following Nadofaragene administration (14, 17).

## Ongoing trials and future prospects

The treatment landscape for patients with non-muscle invasive bladder cancer (NMIBC) is undergoing rapid and transformative evolution. Recent advancements in medical research and technology have led to a dynamic shift in therapeutic options, offering new hope and possibilities for patients facing this challenging condition. Emerging treatments, such as gene therapies like nadofaragene firadenovec, are reshaping the way NMIBC is managed, aiming to provide more effective and targeted interventions. Additionally, the integration of immunotherapies and combination therapies, like pembrolizumab and gemcitabine/docetaxel, respectively, is altering the traditional approach to NMIBC treatment, harnessing the power of the immune system and multi-faceted drug combinations for improved outcomes. These breakthroughs not only expand the array of available therapies but also underline the remarkable progress being made in addressing the unmet medical needs of NMIBC patients, marking a pivotal era of innovation and promise in bladder cancer care. **Table 5** illustrates a diverse range of phase II-IV clinical trials aimed at exploring innovative treatments, combinations of drugs, and methods of administration for NMIBC. These phase II-IV trials showcase the relentless pursuit of improved therapies, innovative drug combinations, and advanced delivery approaches. The breadth of investigations underscores the medical community’s dedication to addressing the challenges posed by NMIBC, particularly in cases that are unresponsive to conventional treatments. As these trials progress and yield valuable insights, they offer the potential to revolutionize the management and outcomes of NMIBC patients, ushering in a new era of more effective and tailored therapeutic interventions.

**Table 5:** Trial data obtained from clinicaltrials.gov.

Study Identifier	Phase	Agents	Primary Endpoint	Estimated Primary Completion Date	Population (n)
NCT04859751	III	VB4-845 Injection in BCG unresponsive pts	Complete response rate	6/2022	53
NCT04165317	III	Sasanlimab + BCG vs BCG alone for induction (+/− maintenance) for high risk NMIBC	Event-free survival	6/2024	999
NCT04490993	III	APL-1202 with Epirubicins hydrochloride vs Epirubicin hydrochloride alone in intermediate and high-risk chemo-refractory NMIBC	Event-free survival	5/2022	359
NCT03982797	II	BCG Moreau strain (not currently authorized) in high risk NMIBC	Progression-free survival	4/2021	306
NCT03528694	III	Durvalumab and Bacillus Calmette-Guerin Combination therapy in high risk NMIBC	Disease-free survival	11/2021	1019
NCT03022825	II/III	Combination BCG with ALT-803 (an IL-15 superagonist) for BCG Unresponsive High Grade NMIBC	Complete response, disease-free rate	1/2023	180
NCT04387461	II	Combination CG0070 (engineered oncolytic adenovirus) + pembrolizumab for BCG unresponsive CIS	Complete response rate	12/2021	37
NCT04172675	II	Erdafitinib (fibroblast growth factor receptor 1–4 inhibitor) vs Intravesical Chemotherapy for high-risk BCG unresponsive pts with FGFR Mutations or Fusions	Recurrence-free survival	10/2022	280
NCT04738630	II	Efficacy and safety of HX008 (humanized anti-PD-1 monoclonal antibody) for BCG-unresponsive NMIBC	Complete response, disease-free survival	12/2022	110
NCT03711032	II/III	Efficacy and safety of pembrolizumab + BCG in high-risk NMIBC for BCG-naive or persistent/recurrent post-BCG Induction	Complete response rate, event-free survival	5/2022	1525
NCT03799835	III	Efficacy of Atezolizumab + one year BCG in BCG-naive Patients With high-risk NMIBC	Recurrence-free survival	4/2022	516
NCT03914794	II	Pemigatinib (FGF receptors 1, 2, and 3 inhibitor) before TURBT for pts with recurrent tumors and prior low or intermediate-risk NMIBC tumors	Complete response rate	5/2022	43
NCT03379909	II	3 months of oral metformin for low-grade NMIBC after TURBT	Overall response	1/2022	49
NCT04452591	III	CG0070 + n-dodecyl-B-D-maltoside (detergent) for BCG unresponsive CIS	Complete response rate	12/2022	110
NCT04736394	III	Oral APL-1202 as a single agent for intermediate-risk NMIBC	Event-free survival	3/2025	800
NCT04386746	II/III	Combination intravesical Gemcitabine and Docetaxel for BCG-naive NMIBC	3-month complete response rate	8/2022	26
NCT04149574	III	Combination Nivolumab + BCG for high-risk BCG that is persistent or recurrent after BCG treatment	Event-free survival	11/2022	700
NCT02371447	I/II	Safety and efficacy of intravesical instillation of VPM1002BC (recombinant BCG) for recurrent NMIBC after TURB and standard BCG therapy	Dose-limiting toxicity, recurrence-free rate	10/2019	39
NCT03560479	I/II	Intravesicular alpha1H prior to transurethral surgery	Safety, efficacy, change in baseline characteristics	12/2021	52
NCT02449239	II/III	Vicinium (active ingredient VB4-845) for high-risk NMIBC after BCG failure	Complete response rate	5/2022	134
NCT04179162	I/II	Combination intravesical Gemcitabine and BCG for BCG-relapsing but responsive HG disease	Maximum tolerated dose, disease-free survival	11/2022	68
NCT04640623	II	Tar200/gemcitabine (intravesicular drug delivery system) with or without Cetrelimab	Clinical response	10/2024	200
NCT04106115	I/II	Durvalumab in combination with S-488210/S-488211 (a 5-peptide cancer vaccine)	Dose-limiting toxicity, Disease Free Survival Rate	8/2024	64
NCT04922047	I/II	Tislelizumab (PD-1 antibody) alone and with BCG for high-risk NMIBC	Dose-limiting toxicity	12/2021	6
NCT03081858	II/III/IV	Proliposomal Intravesical Paclitaxel for Low-Grade NMIBC	Dose-limiting toxicity, Marker lesion response rate	8/2020	15

## Conclusion

In the realm of bladder oncology, the emergence of Nadofaragene firadenovec stands as a watershed moment, symbolizing a transformative breakthrough in the treatment of high-risk, BCG-unresponsive non-muscle invasive bladder cancer (NMIBC). This novel gene therapy, marked by its ingenious design and innovative mechanism of action, holds the promise of reshaping the landscape of NMIBC treatment. With its ability to stimulate interferon production within urothelial cells, Nadofaragene firadenovec orchestrates a multifaceted assault on cancer, invoking immunostimulatory, antiangiogenic, and apoptotic effects. Against the backdrop of the current therapeutic limitations and challenges in addressing high-risk BCG-unresponsive NMIBC, the approval of Nadofaragene firadenovec in December 2022 by the United States regulatory authorities signifies a momentous turning point. Approximately 30% of patients remain refractory to BCG treatment, necessitating alternative therapeutic pathways.

The advent of Nadofaragene firadenovec introduces an optimistic stride towards providing a potent and authorized therapeutic intervention, filling the void in the treatment landscape for these individuals. As the clinical trials of Nadofaragene firadenovec progress and demonstrate its potential efficacy, this groundbreaking therapy brings renewed hope to patients and medical practitioners alike. The evolution of NMIBC treatment is now marked by innovative gene therapies that aim to revolutionize patient outcomes and redefine the approach to bladder cancer therapy. This new era of personalized and targeted interventions holds immense promise, heralding an era of improved clinical management and enhanced quality of life for those affected by high-risk BCG-unresponsive NMIBC.

## Data availability statement

The original contributions presented in the study are included in the article/supplementary material. Further inquiries can be directed to the corresponding author.

## Author contributions

AN, WB and KQ were involved in the study concept, the collection of the data, drafting, literature review, data validation, supervision, and editing of the manuscript. LV, AA and RT were responsible for the literature review and revising the manuscript for important intellectual content. All authors contributed to the article and approved the submitted version.
